# Demographic and clinical characteristics of patients with metastatic breast cancer and leptomeningeal disease: a single center retrospective cohort study

**DOI:** 10.1007/s10549-024-07339-1

**Published:** 2024-06-18

**Authors:** Laura A. Huppert, Samantha Fisch, Elene Tsopurashvili, Sai Sahitha Somepalle, Mia Salans, Harish N. Vasudevan, A. Jo Chien, Melanie Majure, Hope S. Rugo, Ronald Balassanian, Lauren Boreta, Michelle E. Melisko

**Affiliations:** 1 Division of Hematology/Oncology, Department of Medicine, University of California, San Francisco, San Francisco, CA USA; 2 Division of Radiation Oncology, University of California, San Francisco, San Francisco, CA USA; 3 Department of Neurological Surgery, University of California, San Francisco, San Francisco, CA USA; 4 Division of Pathology, University of California, San Francisco, San Francisco, CA USA

**Keywords:** Leptomeningeal disease, Leptomeningeal carcinomatosis, Metastatic breast cancer

## Abstract

**Purpose:**

Leptomeningeal disease (LMD) is a devastating complication of metastatic breast cancer (MBC). It is critical to better understand the risk factors, natural history, and treatment outcomes, including patients in a modern cohort.

**Methods:**

In this single center retrospective cohort study, we identified patients with MBC and LMD who received care from 2000 to 2024 and abstracted key clinical, treatment, and survival data.

**Results:**

We identified 111 patients with MBC and LMD, including patients with the following subtypes: HR+/HER2− (*n* = 53, 47.7%), HER2+ (*n* = 30, 27.0%), and triple negative breast cancer (TNBC; *n* = 28, 25.2%). Median time from the diagnosis of MBC to LMD was 16.4 months (range 0–101.3 months). After the diagnosis of LMD, most patients received systemic therapy (*n* = 66, 59.5%) and/or central nervous system (CNS)-directed therapy (*n* = 94, 84.7%) including intrathecal therapy (*n* = 42, 37.8%) and/or CNS-directed radiation therapy (*n* = 70, 63.1%). In all patients, median overall survival (OS) from the diagnosis of LMD to death was 4.1 months (range 0.1–78.1 months) and varied by subtype, with HR+/HER2− or HER2+ MBC patients living longer than those with TNBC (4.2 and 6.8 months respectively vs. 2.0 months, *p* < 0.01, HR 2.15, 95% CI 1.36–3.39). Patients who received CNS-directed therapy lived longer than those who did not (4.2 vs. 1.3, *p* = 0.02 HR 0.54, 0.32–0.91). Patients diagnosed with LMD from 2015 to 2024 lived longer than those diagnosed from 2000 to 2014 (6.4 vs. 2.9 months, *p* = 0.04, HR 0.67, 95% CI 0.46–0.99). On multivariable analysis, having TNBC was associated with shorter OS from time of LMD to death (*p* = 0.004, HR 2.03, 95% CI 1.25–3.30).

**Conclusion:**

This is one of the largest case series of patients with MBC and LMD. Patients diagnosed with LMD from 2015 to 2024 lived longer than those diagnosed from 2000 to 2014, although median OS was short overall. Patients with TNBC and LMD had particularly short OS. Novel therapeutic strategies for LMD remain an area of unmet clinical need.

## Introduction

One of the most devastating complications of metastatic breast cancer (MBC) is the development of leptomeningeal disease (LMD), also called leptomeningeal carcinomatosis. LMD is defined as cancer cell infiltration into the leptomeninges, including the pia mater, arachnoid, and subarachnoid space from a primary tumor. LMD can be diagnosed based on positive cerebrospinal fluid (CSF) cytology and/or convincing radiographic findings on MRI brain or spinal canal [1]. LMD was once thought to be a rare complication of MBC. However, newer studies show that LMD compromises up to 11–20% of the burden of central nervous system (CNS) disease [2]. With increasing survival after the diagnosis of metastatic disease, as well as improvement in diagnostic imaging and CSF detection techniques, more patients are being diagnosed with LMD over the last 20 years [3]. LMD can cause symptoms including headaches, confusion, word finding difficulties, difficulty with ambulation, incontinence, and other neurologic deficits, which can lead to significant morbidity and very negatively impact quality of life. Prognosis is poor, with a median overall survival of 3–4 months even with aggressive multimodal therapies [4, 5].

Unfortunately, at present there are few effective treatment options for patients with LMD and there is no generally accepted standard of care for patients with LMD. Surgery for placement of a ventriculo-peritoneal shunt (if hydrocephalus is present), radiation therapy, chemotherapy (intravenous or intrathecal) and/or other systemic therapies, and best supportive care can all be considered, and selection of treatment depends upon the patient’s performance status and goals of care. Radiation therapy as a palliative or adjunctive therapy with other systemic therapies may include whole brain radiation therapy (WBRT), craniospinal irradiation, or focal radiation to sites of symptomatic LMD [1]. Intrathecal (IT) therapy including chemotherapies such as IT methotrexate, depot cytarabine, thiotepa or topotecan have been utilized over the past 20 years, although several agents are no longer commercially available and responses are generally limited [6–13]. IT trastuzumab has been studied in patients with human epidermal growth factor receptor 2 (HER2) positive LMD [14–18], including in a recent prospective study in 34 patients with HER2 + MBC and MBC, demonstrating a median overall survival of 10.5 months [19]. In the last decade, several intravenous (IV) or oral agents have been approved for MBC that have superior central nervous system (CNS) penetration. Patients with HER2-overexpressing breast cancers have had more effective systemic therapy for CNS disease since the introduction of lapatinib in 2007 and specifically since the LANDSCAPE trial in 2013, which demonstrated that patients with parenchymal brain metastases could delay radiation and a substantial percentage of intracranial responses were observed [20–22]. Improvements and advances in HER2 targeted TKIs including neratinib, tucatinib, and pyrotinib have improved outcomes for patients with HER2 + MBC with CNS disease [23–25]. Tucatinib in combination with capecitabine and trastuzumab was studied specifically in the treatment of patients with HER2 + LMD in the TBCRC 49 study; in the 17 patients treated, the median time to CNS progression was 6.9 months and median overall survival was 10 months [26]. Most recently, there is some data that the antibody–drug conjugate (ADC) trastuzumab deruxtecan (T-DXd) also has CNS activity [27, 28], and multicenter retrospective data demonstrated responses in patients with MBC and LMD [29]. Therapy advances for patients with LMD and hormone receptor positive (HR+)/HER2− or triple negative breast cancer (TNBC) have been more limited. The CDK4/6 inhibitor abemaciclib was studied in a small cohort of patients (*n* = 10) with HR+/HER2− LMD and no LMD responses were observed [30]. For patients with all breast cancer subtypes, a multicenter phase II study evaluated the use of ANG1005, a brain-penetrating peptide-drug conjugate and showed some activity in patients with breast cancer and LMD and/or recurrent brain metastases [31]. There have also been clinical trials evaluating the safety and efficacy of IV immunotherapy for patients with LMD, although response rates are limited [32, 33]. Unfortunately, patients with LMD after often excluded from clinical trials [34] which has impeded progress, and novel therapies are desperately needed.

Prior retrospective cohort studies have described the diagnosis, treatment, and survival of patients with MBC and LMD, although many of these case series are older and pre-date the introduction of some CNS-penetrant therapies described above. In one of the largest case series to date, Morikawa et al identified 318 patients treated from 1998 to 2013 with LMD and MBC at a single center. In this cohort, 44% of patients had HR+/HER2− MBC, 26% had HER2 + MBC, and 26% had TNBC. Most patients underwent a combination of radiation (64%), IT therapy (46%), and systemic therapy (20%) with an overall median survival of 3.5 months from date of LMD diagnosis [3]. Median overall survival varied across case series, ranging from 2 to 4.5 months, which was typically shorter in patients with TNBC (2–2.5 months) and longer in patients with HER2 + disease (5.2–8.4 months) [3, 35, 36]. There was significant variation among factors that improved overall survival in these case series. Some studies found that receipt of any therapy or a combination of systemic or CNS directed therapy was beneficial to overall survival [37–39]. Other studies specifically found no benefit of radiation without IT or systemic therapy [36, 40]. A few groups collected data on only IT therapy [41] or systemic therapy [35, 42, 43] and demonstrated an improvement in survival compared to no treatment. Finally, one study by Azevedo et al. found no significant impact on survival with any form of treatment studied [44]. However, almost all studies agree that functional status and subtype of disease had a significant association with overall survival after LMD diagnosis [37, 41, 44]. While these data provide important information about the natural history and treatment of LMD, the majority of these retrospective cohort studies, including the larger study done by Morikawa et. al., are based on data primarily gathered from the 1990s and 2000s, and therefore look at outcomes prior to the availability of newer HER2-directed therapies and systemic therapies like antibody–drug conjugates. To our knowledge, the study by Wallace et al. is one of the only more recent studies that evaluated patients with MBC and LMD from 2011 to 2020 and interestingly found similar numbers in median overall survival (4.5 months) compared to older cohort studies [36]. In this study, the authors found that systemic therapy, IT therapy, and WBRT prolonged survival for all patients, and showed that lapatinib and trastuzumab improved overall survival (OS) in patients with HER2 + MBC and LMD [36].

It is critical to better understand the risk factors and natural history of LMD, including patients in a modern cohort after the introduction of additional therapies with improved CNS penetration. In this retrospective cohort study, we identified patients with MBC and LMD at a single center. Using detailed chart extraction, we evaluated demographic and clinical characteristics, treatment history, time to the development of LMD, management strategies, and overall survival.

## Methods

### Study design and objectives

This is a retrospective study of patients with MBC and LMD treated at the University of California San Francisco (UCSF) between 1/2000 and 1/2024. The primary objective of this study is to characterize the clinical characteristics and outcomes of patients with MBC and LMD. Secondary objectives include identifying risk factors associated with poor prognosis.

### Participant identification

Patients were identified in three ways: (1) Via a search of the UCSF pathology database for patients with breast cancer and positive cerebrospinal fluid cytology; (2) Via a search of the UCSF radiation oncology database for patients with MBC and LMD receiving radiation at UCSF; and (3) Via a search of the deidentified form of the UCSF medical record using the Electronic Medical Record Search Engine (EMERSE) platform [45], a search engine that enables investigators to search a complete deidentified copy of the UCSF medical record, using the search terms “breast cancer” and “leptomeningeal disease” or “LMD” or “leptomeningeal carcinomatosis”. Patients were 18 years of age or older. Patients were excluded during initial screening if they were duplicated or if they did not have a primary diagnosis of breast cancer. After chart abstraction, additional patients were excluded if there were insufficient clinical records, insufficient radiology records, and/or if the diagnosis of LMD could not be confirmed.

### Chart abstraction

Detailed information including patient demographics and tumor characteristics, treatment history, lab values, clinical outcomes, and survival dates were obtained via manual chart abstraction. Survival data was updated on 1/15/24.

### Statistics

Data were analyzed using Prism Software (GraphPad; San Diego, CA) and R version 4.2.2 (www.r-project.org). Descriptive statistics were used to summarize numeric responses as rate of events (%) and median (range) as appropriate. Comparisons between groups were made using the unpaired *t*-test, two-sided Fisher’s exact test, or log-rank (Mantel-Cox) test as appropriate. Survival distributions were estimated by the Kaplan–Meier non-parametric method. Univariable and multivariable analyses of factors associated with OS after diagnosis of LMD were evaluated using the Cox proportional-hazards model. All variables with *p* value < 0.1 on univariable analysis were included in the final multivariable model. *p* values of < 0.05 was considered statistically significant.

### Institutional review board statement

This research was approved by the UCSF Institutional Review Board.

## Results

### Patient demographic characteristics and tumor characteristics at MBC diagnosis

111 patients with MBC and LMD were identified at our institution and included in this analysis. Patient demographic and disease characteristics are shown in Table 1. Most patients were female (*n* = 110; 99.1%), non-Hispanic (*n* = 96, 86.5%), and white (*n* = 77, 69.4%). Median age at MBC diagnosis was 51.4 years (range 25.8–80.0 years). Most patients had ductal histology (*n* = 76, 68.5%), but patients with lobular histology or mixed ductal/lobular histology (*n* = 23 and *n* = 2 respectively, 22.5% combined) were over-represented compared to the general MBC population. 23 patients (20.7%) had de novo metastatic disease. Patients had the following MBC subtypes: HR+/HER2− (*n* = 53, 47.7%), HR+/HER2+ (*n* = 24, 21.6%), HR−/HER2+ (*n* = 6, 5.4%), and TNBC (*n* = 28, 25.2%). Most patients had parenchymal brain metastases (*n* = 82, 73.9%) in addition to LMD.

**Table 1:** Patient demographic and disease characteristics

Demographic data (*n* = 111)	
Sex	
Female	110 (99.1%)
Male	1 (0.9%)
Age at diagnosis of MBC	
Median, years	51.4
Range, years	25.8–80.0
Ethnicity	
Hispanic	11 (9.9%)
Non-Hispanic	96 (86.5%)
Unknown/declined	4 (3.6%)
Race	
White	77 (69.4%)
Black	3 (2.7%)
Asian	12 (10.8%)
Native American or Alaska Native	2 (1.8%)
Native Hawaiian or Pacific Islander	1 (0.9%)
Other	14 (12.6%)
Unknown/declined	2 (1.8%)
Tumor characteristics and biology at MBC diagnosis	
Tumor histology	
Ductal	76 (68.5%)
Lobular	23 (20.7%)
Mixed ductal and lobular	2 (1.8%)
Unknown/not specified	10 (9.0%)
Receptor status	
HR + /HER2-	53 (47.7%)
HR + /HER2 +	24 (21.6%)
HR-/HER2 +	6 (5.4%)
TNBC	28 (25.2%)
De novo metastatic disease	
No	88 (79.3%)
Yes	23 (20.7%)
Sites of metastatic disease (at any time point)	
Brain	82 (73.9%)
Bone	71 (64.0%)
Liver	42 (37.8%)
Lung	30 (27.0%)

*HR* hormone receptor, *HER*2 human epidermal growth factor receptor 2, *MBC* metastatic breast cancer, *TBNC* triple negative breast cancer

### Systemic therapy in the metastatic setting prior to the diagnosis of LMD

Systemic therapy in the metastatic setting prior to the diagnosis of LMD is shown in Table 2. Across all breast cancer subtypes, patients received a median of 3 prior lines of therapy for MBC prior to diagnosis of LMD (range 1–10), including a median of 2 prior lines of chemotherapy (range 0–8) and 1 prior line of endocrine therapy (range 0–5). Lines of therapy by breast cancer subtype is also shown in Table 2. Of the patients with HER2+ disease, half received a prior HER2-directed TKI (15/30, 50%). A minority of patients received prior ADC therapy (14/111, 12.6%), likely due to the date range of this retrospective registry before ADC approvals. Similarly, only a minority of patients with TNBC received prior checkpoint inhibitor therapy (3/28, 10.7%).

**Table 2:** Systemic therapy in the metastatic setting prior to the diagnosis of LMD

Systemic therapy for metastatic disease prior to the diagnosis of LMD				
	Overall (*n* = 111)	HR+/HER2− (*n* = 53)	HER2+ (*n* = 30)	TNBC (*n* = 28)
Median lines of therapy for metastatic disease prior to the diagnosis of LMD, median (range)				
Median lines chemotherapy (range)	2 (0–8)	2 (0–8)	2 (0–7)	2 (0–7)
Median lines ET (range)	1 (0–5)	2 (0–4)	1 (0–5)	NA
Median lines total therapy (range)	3 (1–10)	3 (0–10)	2 (0–10)	2 (0–7)
Systemic therapies of interest for metastatic disease prior to the diagnosis of LMD, *n* (%)				
HER2-directed TKIs	15 (13.5%)	NA	15 (50.0%)	NA
Antibody–drug conjugates	14 (12.6%)	2 (3.7%)	9 (30.0%)	3 (10.7%)
T-DM1	9 (8.1%)	NA	9 (30.0%)	NA
T-DXd	2 (1.8%)	2 (3.7%)	NA	NA
Sacituzumab govitecan	3 (2.7%)	0 (0.0%)	NA	3 (10.7%)
Checkpoint inhibitors	5 (4.5%)	2 (3.7%)	NA	3 (10.7%)

*LMD* Leptomeningeal disease, *ET* endocrine therapy, *HR* hormone receptor, *HER*2 human epidermal growth factor receptor 2, *TBNC* triple negative breast cancer, *TKIs* tyrosine kinase inhibitors, *T-DM*1 trastuzumab emtansine, *T-DXd* trastuzumab deruxtecan

### Time from the diagnosis of MBC to the diagnosis of LMD

Time from the diagnosis of MBC to the diagnosis of LMD is shown in Table 3. The median time from the diagnosis of MBC to LMD was 16.4 months (range 0–101.3 months). Median time from MBC to LMD differed by subtype with longer time to LMD in patients with HR+/HER2− (20.5 months) and HER2+BC (17.5 months) vs. shorter time in patients with TNBC (10.5 months) (*p* < 0.01). There was no statistically significant difference in time from MBC to LMD by histology: ductal vs. lobular or mixed ductal/lobular (16.8 vs. 15.8 months, *p* = 0.65) or by year of MBC diagnosis 1999–2014 vs. 2014–2023 (17.0 vs. 15.2, *p* = 0.28). Some patients had progression of systemic disease at the time of LMD diagnosis (*n* = 62, 55.9%), while other patients had stable or improved extracranial disease at the time of LMD diagnosis (*n* = 49, 44.1%).

**Table 3:** Time from the diagnosis of MBC to the diagnosis of LMD

Time from the diagnosis of MBC to the diagnosis of LMD		
	Median (range), months	*p* value
All patients	16.4 (0–101.3)	
By receptor status		
HR+/HER2−	20.5 (0–75.4)	< 0.01 for TNBC vs other subtypes
HER2+	17.5 (0–101.3)	
TNBC	10.5 (0.0–42.4)	
By histology		
Ductal	16.8 (0–101.3)	0.65
Lobular	15.8 (0–75.0)	
By date of MBC diagnosis		
MBC diagnosis 1999–2014	17.0 (0–101.3)	0.11
MBC diagnosis 2015–2024	14.2 (0–69.1)	

*MBC* Metastatic breast cancer, *LMD* leptomeningeal disease, *HR* hormone receptor, *HER*2 human epidermal growth factor receptor 2, *TBNC* triple negative breast cancer

### Systemic and CNS-directed therapy in the metastatic setting after the diagnosis of LMD

Systemic and CNS-directed therapy in the metastatic setting after the diagnosis of LMD is shown in Table 4. After the diagnosis of LMD, most patients received systemic therapy (*n* = 66, 59.5%) with a median of 1 line of systemic therapy (range 0–4). The percentage of patients who received any systemic therapy after the diagnosis of LMD differed by subtype: HR+/HER2− (33/53, 62.3%), HER2+(22/30, 73.3%), TNBC (11/28, 39.3%).

**Table 4:**
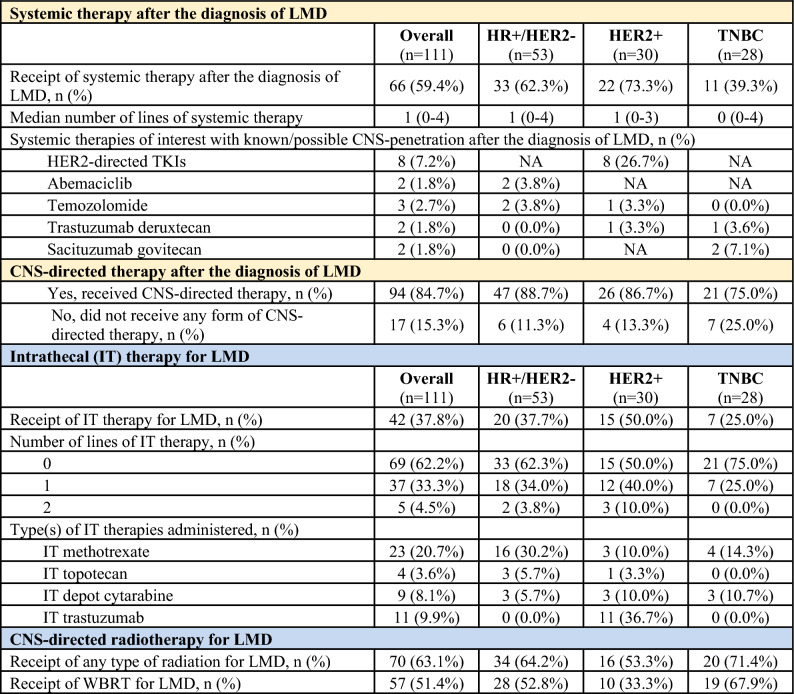
Systemic and CNS-directed therapies in the metastatic setting after the diagnosis of LMD

After the diagnosis of LMD, most patients also received CNS-directed therapy (*n* = 94, 84.7%), including intrathecal therapy (*n* = 42, 37.8%) and/or CNS-directed radiation therapy (*n* = 70, 63.1%). Rates of IT therapy use varied by subtype: HR+/HER2− (20/53, 37.7%), HER2+(15/30, 50.0%), TNBC (7/28, 25.0%). Of the 42 patients to have received IT therapy, most received 1 line of IT therapy (*n* = 37, 88.1%) but some received 2 lines of IT therapy (*n* = 5, 11.9%). Types of IT therapies administered included IT methotrexate (*n* = 23, 20.7%), IT topotecan (*n* = 4, 3.6%), IT depot cytarabine (*n* = 9, 8.1%), and/or IT trastuzumab (*n* = 11, 9.9%). Most patients received CNS-directed radiation for LMD (*n* = 70, 63.1%), including many who received WBRT (*n* = 57, 51.4%).

A minority of patients received no therapy after the diagnosis of LMD (neither systemic nor CNS-directed therapy) (*n* = 17, 15.3%).

### Overall survival from diagnosis of LMD to death

**Fig. 1:**
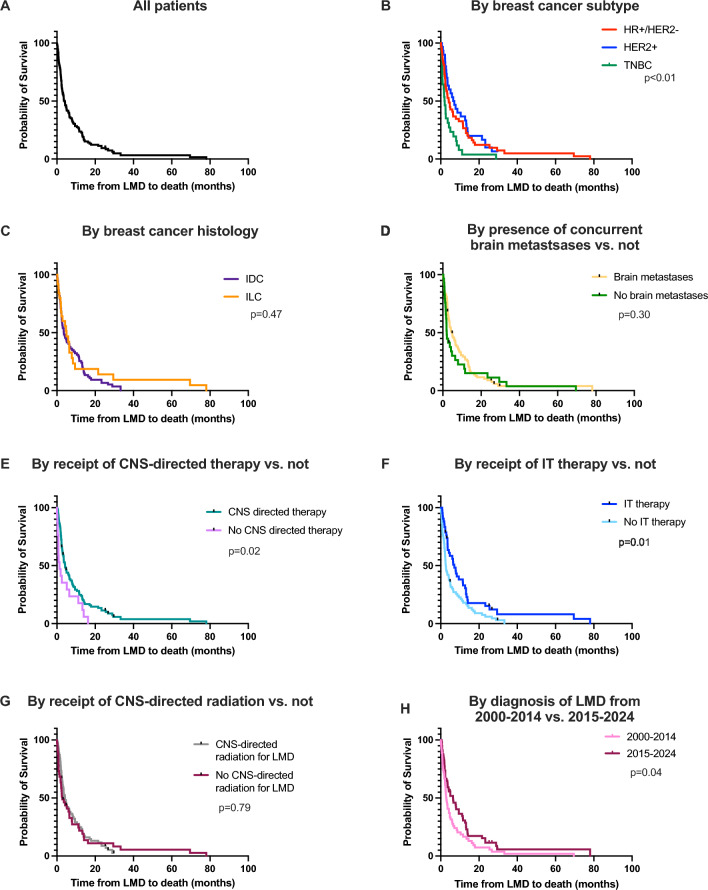
Overall survival from the diagnosis of LMD to death. Kaplan–Meier curves showing survival data of patients in this cohort from diagnosis of LMD (leptomeningeal disease) to death: **A** For all patients in this cohort (*n* = 111). **B** By breast cancer subtype: HR+/HER2− (red, *n* = 53), HER2+ (blue, *n* = 30), vs. TNBC (green, *n* = 28). **C** By breast cancer histology: invasive ductal carcinoma (IDC) (purple, *n* = 76) vs. invasive lobular carcinoma (ILC) (orange, *n* = 23). **D** By presence of concurrent parenchymal brain metastases (yellow, *n* = 82) vs. no parenchymal brain metastases (green, *n* = 29). **E** By receipt of CNS-directed therapy (teal, *n* = 94) vs. no receipt of CNS-directed therapy (pink, *n* = 17). **F** By receipt of intrathecal (IT) therapy (dark blue, *n* = 42) vs. no IT therapy (light blue, *n* = 69). **G** By receipt of central nervous systemic (CNS)-directed radiation for LMD (gray, *n* = 70) vs. no CNS-directed therapy for LMD (magenta, *n* = 41). **H** By diagnosis of LMD from 2000 to 2014 (light pink, *n* = 57) vs. 2015–2024 (dark pink, *n* = 54). (Color figure online)

Kaplan–Meier curves for survival data from the diagnosis of LMD to death are shown in Fig. 1 and favors associated with OS in univariate and multivariate analysis are shown in Table 5. In all patients, median OS from the diagnosis of LMD to death was 4.1 months (range 0.1–78.1 months) (Fig. 1A). Forty-three patients were alive at least 6 months (38.7%), 25 patients were alive at least 12 months (22.5%), and 10 patients were alive at least 24 months (9.0%). Median OS from LMD to death varied by subtype, with patients with HR+/HER2− or HER2+MBC and LMD living longer than those with TNBC and LMD (4.2 and 6.8 months respectively vs. 2.0 months, *p* < 0.01, HR 2.15, 95% CI 1.36–3.39) (Fig. 1A). There was no significant difference in median OS by histology: ductal vs. lobular (3.6 vs. 5.0 months, *p* = 0.47, HR 0.83, 95% CI 0.50–1.37) (Fig. 1C) or by presence vs. absence of concurrent brain metastases (4.7 vs. 2.0 months, *p* = 0.30, HR 0.79, 95% CI 0.51–1.23) (Fig. 1D). Patients who received CNS-directed therapy survived longer than those who did not receive CNS-directed therapy (4.2 vs. 1.3 months, *p* = 0.02, HR 0.54, 95% CI 0.32–0.91) (Fig. 1E). Patients who received IT therapy survived longer than those who were not treated with IT therapy (7.1 vs. 2.7, *p* = 0.01, HR 0.60, 95% CI 0.40–0.90) (Fig. 1F), whereas there was no statistically significant difference in survival based on receipt of CNS-directed radiation vs. not (4.2 vs. 2.8, *p* = 0.79, HR 0.95, 95% CI 0.63–1.43) (Fig. 1G). Patients diagnosed with LMD from 2015 to 2024 lived longer than those diagnosed from 2000 to 2014 (6.4 vs. 2.9 months, *p* = 0.04, HR 0.67, 95% CI 0.46–0.99) (Fig. 1H). On multivariable analysis, having TNBC was associated with shorter OS from time of LMD to death (*p* = 0.004, HR 2.03, 95% CI 1.25–3.30).

**Table 5:** Factors associated with overall survival from the diagnosis of LMD to death in univariable and multivariable analysis

Characteristic	Univariable			Multivariable		
	Hazard ratio	95% CI	*p* value	Hazard ratio	95% CI	*p* value
Age at LMD (continuous)	1.00	0.98–1.02	0.96			
Race						
White	Ref	–	–			
Non-white	1.11	0.72–1.69	0.64			
Hispanic/Latino	0.50	0.24–1.04	0.06	0.69	0.32–1.50	0.36
De novo metastatic disease	0.81	0.51–1.3	0.34			
Subtype						
HR + /HER2-	Ref	–	–	Ref	–	–
HER2 +	1.10	0.48–2.54	0.82	1.07	0.40–2.18	0.88
TNBC	2.15	1.36–3.39	**0.001**	2.03	1.25–3.30	**0.004**
Histology						
Ductal	Ref	–	–			
Lobular	0.83	0.50–1.37	0.47			
Mixed	1.04	0.14–7.60	0.97			
Unknown	1.00	0.50–2.01	1.00			
Brain metastases	0.79	0.51–1.23	0.30			
CNS-directed therapy	0.54	0.32–0.91	**0.02**			
IT chemotherapy	0.60	0.40–0.90	**0.01**	1.38	0.47–1.11	0.14
CNS-directed radiation	0.95	0.63–1.43	0.79			
Date of LMD diagnosis						
2000–2014	Ref	–	–	Ref	–	–
2015–2024	0.67	0.46–0.99	**0.04**	0.70	0.46–1.07	0.10

All variables with *p* value < 0.1 on univariable analysis were included on multivariable analysis except for receipt of CNS-directed therapy (which includes both receipt of IT chemotherapy and CNS-directed radiation therapy)

*CI* Confidence interval, *LMD* leptomeningeal disease, *HR* Hormone receptor, *HER2* human epidermal growth factor receptor 2, *TBNC* triple negative breast cancer, *CNS* central nervous system, *IT* intrathecal

Bold indicates stastical significance with p-value <0.05

## Discussion

This study represents one of the largest reported case series of patients with MBC and LMD, including a more modern cohort with most patients diagnosed after 2010. Through detailed chart abstraction, we describe the patient demographic characteristics, disease biology, treatment history before and after the diagnosis of LMD, and overall survival in these patients.

In our cohort, we found that patients with HER2 + (27.0%) and TNBC (25.2%) were over-represented compared to the overall population of patients with MBC (typically 10–15% for each subtype) [46], which is consistent other published data [3, 37, 39, 41, 42]. Multiple mechanisms have been reported to explain the increased incidence of CNS disease in HER2-overexpressing cancers, both in mouse models and in humans [47]. Biological factors that may predispose patients with TNBC to preferentially developing CNS disease include an increased epithelial-to-mesenchymal transition (EMT) phenotype, “stem cell like” behavior, and upregulation of transcriptions factors such as MYC [48]. We also observed that patients with invasive lobular histology (22.5%) were overrepresented compared to the general population (10–15%) [49], which has also been reported in other LMD case series. Biologically, this is consistent with the behavior of lobular cancers which, due to loss of e-cadherin, a transmembrane glycoprotein that mediates a cell–cell adhesion, have a predisposition to spread to mucosal surfaces such as the peritoneum and GI tract [50]. The relatively young median age of patients and high percentage of patients with de novo metastatic disease may reflect the referral pattern of younger, healthier patients to our academic center. Consistent with prior studies, most patients in our cohort had concurrent brain metastases and LMD [3, 36, 43].

Prior to the diagnosis of LMD, patients in our cohort received a median of three prior lines of systemic therapy. In this more modern cohort of patients, we were specifically interested in whether patients received prior therapies known to have improved CNS penetration. We found that half of patients with HER2 + disease had received prior HER2−targeted TKIs. Fewer patients had received ADCs prior to the diagnosis of LMD, likely due to the more recent approvals of these agents. Across all patients, the median time from the diagnosis to MBC to the diagnosis of LMD was 16.4 months, with a wide range from 0.0 months (LMD at time of MBC diagnosis) up to 101.3 months. This is similar to prior retrospective studies, such as the median time from MBC to LMD of 22.5 months in the study by Morikawa et al. and 22.0 months in the study by Gauthier et al. [3, 40]. Of note, there is variation in the frequency of CNS imaging and when radiologists make the diagnosis of LMD, so it is possible that the slightly shorter time from MBC to LMD in our population may have been due to more frequent CNS-imaging in our academic, clinical trial population, although this was not formally evaluated. Time from the diagnosis of MBC to the diagnosis of LMD was longer for patients with HR+/HER2− and HER2 + disease than for TNBC but did not differ by histology. Interestingly, there was no difference in time from MBC to LMD diagnosis by year of MBC diagnosis from 1999 to 2014 vs. 2015–2023. With tremendous advances in general for patients with MBC in the last decade, this may be due to the small sample size and random variance in this cohort, or it could speak to the fact that many agents do not have sufficient CNS penetration, so while patients with MBC in general are living longer, we may not be adequately preventing the development of LMD. Of note, about one half of patients were diagnosed with LMD at the time of systemic disease progression, whereas the other half of patients had stable/responding disease at the time of LMD diagnosis, which underlies the fact that the CNS compartment and in particular the CSF tumor microenvironment may be distinct from the systemic tumor microenvironment [51].

After the diagnosis of LMD, patients received a median of one additional line of systemic therapy, including 25% of patients with HER2 + breast cancer who continued or were treated with a HER2-directed TKI. Most patients also received some form of CNS-directed therapy, including IT therapy and/or CNS-directed radiotherapy. Specifically, 37.8% of patients received IT therapy, most commonly IT methotrexate for patients with HR+/HER2− and TNBC LMD and IT trastuzumab for patients with HER2 + LMD. Most patients also received radiation for LMD, including over half of the patients being treated with WBRT.

Among the patients with MBC and LMD in this cohort, the median OS from the diagnosis of LMD to death was 4.1 months, which varied by subtype: HR+/HER2− 4.2 months, HER2 + 6.8 months, and TNBC 2.0 months. These survival data are consistent with prior studies of MBC and LMD. For example, in a retrospective study of 318 patients with MBC and LMD from 1998 to 2013, the patients in their cohort had a median overall survival of 3.5 months from LMD to death, with subtype variation as follows: HR+/HER2− 3.9 months, HER2 + 5.2 months, and TNBC 2.5 months [3]. In a more recent study by Wallace et al. which included patients from 2011 to 2000, median overall survival from LMD to death by subtype was: HR+/HER2− 5.3 months, HER2 + 8.4 months, TNBC 2.0 months. We observed no difference in survival by histology (ductal vs. lobular), which is consistent with a prior retrospective analysis by Niwinska et al. [38]. Interestingly, there was also no difference in survival by the presence or absence of concurrent brain metastases. We found that patients who received CNS-directed therapy lived longer than those who did not receive CNS-specific therapy, and specifically patients who received IT therapy lived longer than those who did not receive IT therapy, as was also seen in the study by Wallace et al. [36], whereas there was no difference in survival based on receipt of CNS-directed radiation therapy in our cohort. It is possible that IT therapy indeed prolonged OS for these patients, or more likely that patients who had advanced LMD or a poor performance status may not have been offered or chosen to pursue IT therapy. Patients diagnosed with LMD from 2015 to 2024 lived longer than those diagnosed from 2000 to 2014 (6.4 vs. 2.9 months, *p* = 0.04, HR 0.67, 95% CI 0.46–0.99) which may suggest some improvement in outcomes with novel CNS-penetrant agents in a more modern cohort, but survival was still short in both groups and this was not significant on multivariate analysis, so clearly better therapeutic strategies are needed. It is interesting to note that there was a small subset of patients who did live longer, with ten patients in our cohort (9.0%) that survived more than 24 months after the diagnosis of LMD. Further work is needed to determine predictors of favorable outcomes and whether there are one or more biomarkers that can predict longer-term survival.

This study has several notable strengths. First, we performed extensive chart abstraction to capture detailed demographic, clinical, and treatment data. This allowed us to provide a thorough description of the clinical and biologic characteristics of patients who developed LMD and their previous and subsequent treatment courses. Second, the patient population included in this investigation spans a long period of time during which several major therapeutic breakthroughs (ADCs and immunotherapy) were introduced into breast cancer care. The outcomes from this dataset provide important prognostic information for clinicians and patients, including analysis by various subgroups of interest.

This study also has several limitations due to its retrospective nature. First, the absence of a control arm that includes patients with MBC who do not develop LMD limits our ability to interpret specific risk factors for LMD. Subsequent studies are needed to better address this question. Second, reflective of current clinical practice, not all patients underwent evaluation of CSF cytology and complete imaging of the neuro-axis, so we were not able to evaluate survival based on presence or absence of specific cytologic features or radiographic findings, as this data was not available for all patients. Third, while this analysis includes a more modern cohort of patients, a relatively small number of patients received HER2-directed TKIs, ADCs, and other novel therapies prior to and after the diagnosis of LMD, even after some of these agents were approved. This is in part explained by the fact that while patients with HER2 + and triple negative disease are over-represented in this sample, most patients with LMD in our series had HR+/HER2− disease and no systemic targeted therapy has demonstrated efficacy in treatment of LMD. Looking ahead, with more evidence for CNS penetration with some ADCs and many TKIs, and with approval of these agents for ER + and HER2 “low” disease, we may observe better outcomes in these subsets of patients. Fourth, while we found that patients who received CNS-directed therapy and specifically IT therapy lived longer, this is likely confounded by the fact that patients with advanced LMD and/or poor performance status may not have been offered or chosen to pursue additional therapies, so these findings should be interpreted with caution. Finally, retrospective chart reviews are limited by written details included in the clinical record, so it is possible that certain demographic features or aspects of treatment history were not captured if they were not well-documented in the UCSF electronic medical record.

In summary, this retrospective case series helps better define the clinical characteristics, treatment history, and overall survival in a more modern cohort of patients with LMD and MBC. Patients diagnosed with LMD from 2015 to 2024 lived longer than those diagnosed from 2000 to 2014, although survival remained short even in this modern cohort. Novel therapeutic strategies for patients with MBC and LMD remains an important area of unmet clinical need.

## Data Availability

The datasets generated during the current study are not publicly available in order to protect patient privacy.
